# Effect of cooking temperature and time on total phenolic content, total flavonoid content and total in vitro antioxidant activity of garlic

**DOI:** 10.1186/s13104-020-05404-8

**Published:** 2020-12-14

**Authors:** Thandiwe Alide, Phanice Wangila, Ambrose Kiprop

**Affiliations:** 1 Department of Chemistry and Biochemistry, School of Sciences and Aerospace Studies, Moi University, Eldoret, Kenya; 2 Africa Center of Excellence II in Phytochemicals, Textile and Renewable Energy, Moi University, Eldoret, Kenya; 3 Department of Applied Sciences, Malawi Institute of Technology, Malawi University of Science and Technology, Thyolo, Malawi; 4 Department of Physical Sciences, School of Science and Technology, University of Kabianga, Kericho, Kenya

**Keywords:** *Allium sativum*, Fourier transform infrared spectroscopy, Condiment, Allicin, Radical scavenging activity

## Abstract

**Objective:**

To investigate the effect of cooking temperature and time on the total phenolic content, total flavonoid content and antioxidant activity of aqueous and ethanolic extracts of garlic.

**Results:**

The mean total phenolic content of fresh garlic were 303.07 ± 6.58 mg gallic acid equivalent per 100 g (GAE/100 g) and 638.96 ± 15.30 mg GAE/100 g of plant material for the aqueous and ethanolic extracts respectively. The mean total flavonoid content 109.78 ± 6.78 mg quercetin equivalent per 100 g (QE/100 g) and 258.47 ± 12.37 QE/100 g for aqueous and ethanolic extracts respectively. Fourier transform infrared spectral data showed absorptions in the range for carboxylic acids, hydroxyl group, esters, and alcohols, confirming the presence of phenols and flavonoids in the extracts. Cooking temperature had a significant effect on total phenolic content and total flavonoid content while cooking time did not have a significant effect on the phytochemicals and antioxidant activity.

## Introduction

*Allium sativum* L. (garlic) is a popular culinary herb due to its aroma and therapeutic properties [1, 2]. Food additives such as garlic are known to possess antimicrobial activities and provides protection against degenerative diseases [2, 3]. These bioactivities are attributed to the presence of antioxidants [1, 3–5] that scavenge free radicals in our bodies [6]. Free radicals are molecular species that contain an unpaired electron which makes them highly unstable and reactive and thus act as oxidants [7]. Free radicals in our bodies are as a result of metabolic processes, exposure to sun rays, smoking and exposure to environmental pollutants [8]. There are antioxidant systems within our bodies which are responsible for counteracting the effects of the free radicals. The free radicals could be the cause of the rise in cancer cases which are becoming a concern in Kenya and the world as a whole. Phytochemicals of garlic are promising candidates for cancer therapy [2]. Vitamins A and B, and β-carotene are the principal antioxidant micronutrients in humans but are not synthesized in the body hence they are supplied in the diet. Direct consumption of raw food additives is limited due to their taste, aromatic and pungent properties and therefore often added to food and cooked as whole spices, chopped, powder or extracts [1]. The conditions under which food is prepared will affect its medicinal and nutritive value [1, 9], hence this study. Information on the effect caused by cooking conditions is lacking yet it is crucial in predicting the nutritive value and therapeutic properties of these food additives. The current study investigated the effect of some cooking conditions on the total phenolic content, total flavonoid content and antioxidant activity of garlic.

## Main Text

### Sampling and analysis

Garlic bulbs (5 kg) were purchased from Khethia supermarket in Eldoret town, Kenya. A measured 100 g of chopped garlic (2 mm × 2 mm size) was used. Cooking was done at 25  °C, 50, 75, 100, 125 and 150 °C in 100 ml of water. The samples were heated for 15, 30, 45 and 60 min in each case. The solutions were cooled and filtered using Whatman No.1 filter paper. The filtrates (aqueous extracts) were kept while the residues were further macerated in 50 ml of absolute ethanol for 24 h after which the extracts were filtered. All the extracts were concentrated under reduced pressure at 50 °C on a rotary evaporator. The concentrated extracts were collected in petri dishes and air-dried. In total, 50 samples (25 aqueous and 25 ethanolic extracts) were obtained and stored at 4 °C.

The TPC of the samples was determined using Folin-Ciocalteu reagent [10] using gallic acid as the standard solution [11, 12]. TFC of the extracts was determined using Aluminium Chloride colorimetric method using quercetin as a standard and the results were expressed in mg quercetin equivalent (QE/mg) [13]. All spectroscopic measurements were done using UV-1900 UV–Vis Spectrophotometer (Shimadzu Corporation, Japan). Antioxidant activity was evaluated using 2, 2-diphenyl-1-picrylhydrazyl (DPPH) scavenging assay [14, 15]. Fourier Transform Infrared (FT-IR) analysis was used to confirm the functional groups of the compounds in the extracts [16]. A FT-IR spectrometer (Nicolet NEXUS 470, Thermo Scientific, USA) was used for analysis of the dried powder of the extracts following the method described by Ashokkumar and Ramaswamy [17].

### Statistical analysis

Analytical experiments were run in triplicate and data presented as means ± standard deviations. One-way ANOVA was performed, and the means were separated using the Least Significant Difference post hoc test at *p* < *0.05* using IBM SPSS Statistics v20 (IBM Inc., USA).

### Results

**Fig. 1:**
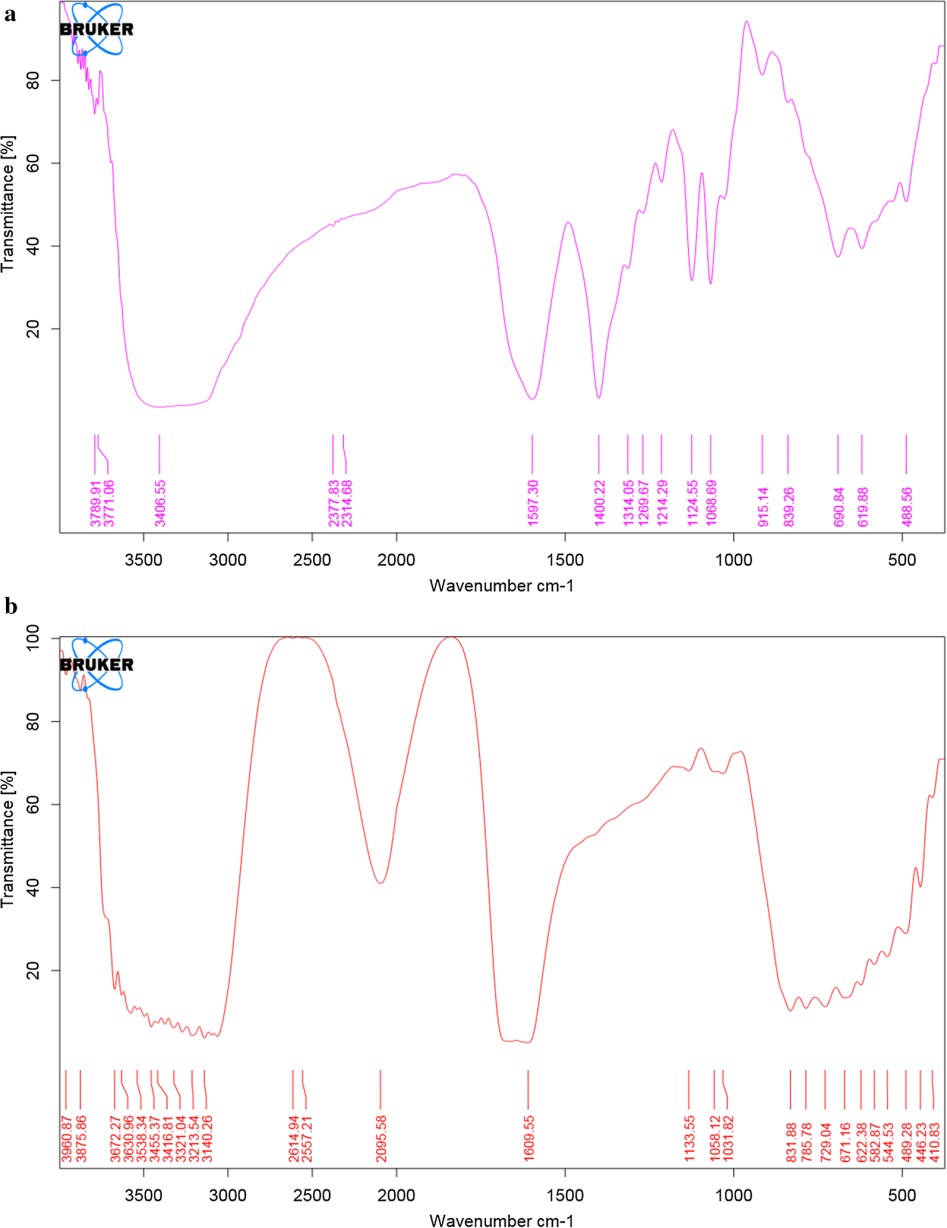
FT-IR spectrum of fresh garlic **a** aqueous, **b** ethanolic extracts

Aqueous and ethanolic extracts of fresh garlic had TPC and TFC of 303.07 ± 6.58 and 638.96 ± 15.30 mg GAE/100 g of plant material and 109.78 ± 6.78 and 258.47 ± 12.37 mg QE/100 g mg respectively. The FT-IR spectra of the fresh extracts is shown in Fig. 1. Table 1 shows the TPC and TFC of the extracts after boiling at the different cooking temperatures and times while the antioxidant activity of the extracts cooked at different temperatures and times are given in Table 2 (Additional file 1: Table S1).

**Table 1:** Total phenolic and total flavonoid contents of the garlic extracts at different cooking temperatures and times

Extract	Time (minutes)	25 $^\circ{\rm C}$	50 $^\circ{\rm C}$	75 $^\circ{\rm C}$	100 $^\circ{\rm C}$	125 $^\circ{\rm C}$	150 $^\circ{\rm C}$
Total phenolic content (mg GAE/100 g)							
Aqueous	15	365.49 ± 13.61^a^	517.28 ± 9.15^b^	701.39 ± 20.71^b^	753.68 ± 13.07^b^	878.65 ± 9.83^c^	933.60 ± 23.41^c^
	30	367.08 ± 14.71^a^	632.86 ± 18.34^b^	753.91 ± 7.42^b^	797.67 ± 17.36^b^	973.775 ± 18.12^c^	1016.86 ± 4.18^c^
	45	369.48 ± 21.48^a^	793.75 ± 33.21^b^	880.43 ± 6.55^b^	768.47 ± 17.71^b^	1091.46 ± 15.75^c^	1199.95 ± 20.78^c^
	60	376.68 ± 20.04^a^	798.52 ± 20.87^b^	897.36 ± 10.51^b^	932.66 ± 13.29^b^	1200.87 ± 39.92^c^	1273.30 ± 17.87^c^
Ethanolic	15	394.42 ± 10.86^a^	381.00 ± 4.58^a^	385.90 ± 5.01^a^	379.02 ± 6.53^a^	293.83 ± 10.65^c^	266.34 ± 1.23^c^
	30	377.95 ± 14.47^a^	382.51 ± 10.17^a^	387.66 ± 7.72^a^	370.20 ± 4.96^a^	285.30 ± 17.52^c^	266.42 ± 9.43^c^
	45	383.80 ± 1.24^a^	381.20 ± 5.38^a^	375.46 ± 9.51^a^	290.00 ± 5.06^a^	255.18 ± 17.94^c^	234.66 ± 12.86^c^
	60	355.31 ± 9.88^a^	367.40 ± 8.22^a^	352.72 ± 13.22^a^	272.77 ± 10.74^a^	230.48 ± 22.40^c^	141.64 ± 3.72^c^
Total flavonoid content (QE/g)							
Aqueous	15	133.82 ± 13.09^a^	148.85 ± 15.74^ab^	229.64 ± 8.50^bc^	246.14 ± 6.47^ cd^	379.54 ± 9.08^de^	374.13 ± 15.43^e^
	30	136.62 ± 7.95^a^	211.47 ± 12.50^ab^	246.87 ± 7.63^bc^	309.25 ± 0.00^ cd^	395.66 ± 12.09^de^	367.11 ± 9.63^e^
	45	165.02 ± 8.37^a^	253.72 ± 6.30^ab^	290.39 ± 21.17^bc^	374.39 ± 4.48^ cd^	392.10 ± 15.46^de^	490.36 ± 10.99^e^
	60	179.61 ± 3.57^a^	268.79 ± 8.79^ab^	296.62 ± 18.37^bc^	374.92 ± 14.72^ cd^	392.28 ± 1.41^de^	522.57 ± 15.59^e^
Ethanolic	15	437.92 ± 4.99^a^	392.78 ± 1.56^b^	324.57 ± 7.06^c^	252.56 ± 13.29^d^	125.54 ± 7.53^e^	125.07 ± 3.71^f^
	30	444.27 ± 3.41^a^	347.34 ± 7.41^b^	291.27 ± 5.79^c^	246.02 ± 8.13^d^	183.89 ± 20.96^e^	86.37 ± 17.08^f^
	45	428.96 ± 1.58^a^	349.19 ± 6.99^b^	274.27 ± 4.17^c^	192.05 ± 9.35^d^	182.55 ± 20.33^e^	71.95 ± 4.02^f^
	60	414.98 ± 20.16^a^	324.53 ± 11.08^b^	247.75 ± 9.54^c^	180.61 ± 4.39^d^	129.26 ± 2.94^e^	69.10 ± 6.03^f^

Values with different alphabetical letters for an extract are statistically different (*p < 0.05*)

**Table 2:** Antioxidant activity of garlic at different cooking temperatures and times

Extract	Time (minutes)	25 $\boldsymbol{^\circ{\rm C} }$	50 $\boldsymbol{^\circ{\rm C} }$	75 $\boldsymbol{^\circ{\rm C} }$	100 $\boldsymbol{^\circ{\rm C} }$	125 $\boldsymbol{^\circ{\rm C} }$	150 $\boldsymbol{^\circ{\rm C} }$
Aqueous	15	42.90 ± 0.72^a^	50.26 ± 0.47^b^	60.50 ± 4.12^bc^	67.97 ± 0.30^ cd^	72.31 ± 0.27^d^	73.95 ± 3.90^d^
	30	48.99 ± 4.50^a^	52.13 ± 0.61^b^	59.42 ± 1.22^bc^	71.38 ± 0.18^ cd^	75.00 ± 2.06^d^	75.75 ± 2.12^d^
	45	48.06 ± 0.79^a^	65.88 ± 0.44^b^	66.22 ± 0.32^bc^	71.52 ± 0.10^ cd^	75.90 ± 1.77^d^	76.98 ± 0.40^d^
	60	48.02 ± 0.55^a^	66.07 ± 3.74^b^	72.80 ± 0.19^bc^	71.82 ± 0.57^ cd^	76.31 ± 0.69^d^	81.95 ± 0.68^d^
Ethanolic	15	48.95 ± 1.22^a^	48.77 ± 0.49^a^	46.04 ± 1.34^ab^	46.64 ± 0.57^b^	31.05 ± 0.45^c^	3.33 ± 1.70^d^
	30	47.68 ± 0.31^a^	49.14 ± 0.95^a^	46.49 ± 0.28^ab^	46.79 ± 0.46^b^	31.39 ± 0.91^c^	2.13 ± 1.26^d^
	45	47.53 ± 0.74^a^	47.27 ± 0.22^a^	47.20 ± 0.79^ab^	27.73 ± 0.37^b^	16.74 ± 2.82^c^	2.24 ± 1.22^d^
	60	37.97 ± 1.84^a^	46.97 ± 4.57^a^	32.03 ± 6.46^ab^	27.58 ± 0.37^b^	15.25 ± 3.16^c^	2.24 ± 0.80^d^

Values with different alphabetical letters for an extract are statistically different (*p < 0.05*)

### Discussion

The TPC of the ethanolic extract of fresh garlic was 638.96 ± 15.30 mg GAE/100 g and that of the aqueous extract was 303.07 ± 6.58 mg GAE/100 g which is 52.7% less than that of ethanolic extract. This indicated that ethanol is a better solvent than water. Similarly, the TFC of ethanolic extract of fresh garlic was 258.47 ± 12.37 mg QE/100 g and that of aqueous extract was 109.78 ± 6.78 mg QE/100 g, 57.5% lower than for ethanolic extract. Mishra et al. [5] reported a TPC of 78.45 mg GAE/100 g of plant material for fresh garlic, which was eight times lower than the TPC reported in this study. Chan et al. [11] found that the TPC of fresh garlic was 154 ± 10 mg GAE/100 g and TFC was 8.3 ± 0.6 mg CE/100 g. A higher TPC (32.17 mg GAE/g) in garlic was reported by Akan [18]. The variations in the findings may be due to the differences in the cultivars, location, climate and maturation of the garlic samples [18].

FTIR analysis for both the garlic extracts showed an absorption band in the range 3400–2400 cm^−1^ (Fig. 1) which is characteristic of carboxylic acids. Peaks were also identified in the range 3650–3600 cm^−1^ for free O–H; bonded O–H at 3400–3200 cm^−1^; C−O around 1300–1000 cm^−1^ for esters, carboxylic acids and alcohols; S = O for sulphoxides at around 1050 cm^−1^ [19]. The observed functional groups are associated with phenolic and flavonoid compounds. Divya et al. [20] reported that the functional groups of garlic showed a peak at 3265 cm^−1^ which was due to O–H stretching of a hydroxyl group. This indicated the presence of polyhydroxy compounds such as flavonoids. A peak at 2926 cm^−1^ was due to asymmetric stretching of C−H groups of aromatic compounds and at 1619 cm^−1^ which was due to C = O stretching of peptide linkages or stretching of carbonyl and carboxylic groups. Another peak at 1395 cm^−1^ was as a result of O–H bend of carboxylic acids whereas a peak at 1036 cm^−1^ was due to S = O for the presence of organosulphur compounds.

### Effect of cooking temperature on total phenolic and total flavonoid contents

The TPC of aqueous extracts was observed to increase with increase in temperature (Table 1). On the contrary, the TPC from the garlic residues macerated with ethanol decreased with increase in cooking temperature. Under the hypothesis that TPC would not vary at different cooking temperatures, there were some significant differences (*p* < *0.05*) in TPC at the different cooking temperatures. These differences were more pronounced for extracts cooked at the temperature extremes. For instance, TPCs recorded at 25 °C were significantly different (*p* < *0.*05) from those recorded at the other temperatures. However, TPCs recorded at 50 °C were not significantly different from those cooked at 75 °C (*p* = *0.148*). This means that increase in cooking temperature increased the TPC of garlic, probably by allowing bound phenolic compounds to be released in the water used for cooking. This statement is corroborated by results which showed that the TPC of the cooked garlic residues (ethanolic extracts) decreased with increasing temperature. Likewise, ethanolic extracts had significant differences in their TPCs at the experimental temperatures used for cooking. Further, marked differences in TPC were noted between ethanolic extracts cooked at high temperatures and those cooked at low temperatures. For example, significant differences (*p* < *0.05*) were observed between residues from 25 °C aqueous extract and those of 125 °C and 150 °C unlike those that were subjected to 75 °C (*p* = *0.925*) and 100 °C (*p* = *0.067*).

Like TPC reported for garlic extracts, there was a general increase in the TFC of aqueous garlic extracts with increase in the cooking temperature. These variations in TFC at different temperatures for aqueous extracts were significant (*p* < *0.05*). For example, the TPC of aqueous extracts boiled at 25 °C significantly differed from those boiled at 75 °C (*p* = *0.005*) and 150 °C (*p* = *0.01*). On the contrary, the TFC of ethanolic extracts decreased as the temperature increased. There were noticeable differences in the TPC of the ethanolic extracts boiled at different temperatures. The increase in TPC following heating is because cooking inactivates polyphenol oxidase enzyme, inhibiting polyphenolics degradation [4].

The results of the current study is comparable to that of Shaimaa et al. [4] who reported that TPC and TFC of sweet and chilli pepper increased after boiling, with the antioxidant activity exhibiting a positive relationship with TPC and TFC. Mishra et al. [5] found that the TPC of garlic after boiling reduced by 34.18–52.87 mg/100 g. This could be because only boiled garlic residues were analyzed leaving behind the phytochemicals in the aqueous extract obtained after boiling unaccounted for. Rupasinghe et al. [21] reported that baking process affected all phenolic compounds and that anthocyanin in apple skin, cyadinin-3-O-galactoside was relatively the most affected in comparison to flavanols, dihydrochalcones, phenolic acids, and flavan-3-ols. A significant rise was also recorded for quercetin and phloretin due to thermohydrolysis during the baking process. According to Benner et al. [22], boiling treatment did not only improve the extraction rate of anthocyanins and other phenolic compounds but also led to their degradation. However, dry heating caused more degradation as compared to boiling. The major degradation products during boiling were protocatechuic acid (phenolic acid) and catechin (flavonoid). These degradation products could also explain why there was an increase in the total phenolic content and total flavonoid content as the cooking temperature was increased in this study.

In addition, the increase or decrease in the phytochemical content of vegetables depends on the type of cooking that was used to determine the effect of temperature and sometimes the type of vegetable. Sedat et al. [23] reported that the TPC of broccoli, pepper and green beans significantly increased by different levels depending on the cooking method and insignificant increase was observed for spinach. On the other hand, the TPC of squash, peas and leek significantly reduced by the same level in all cooking methods. On the same, Wen et al. [24] communicated that the total phenolic content of vegetables increased and for some vegetables it decreased when blanched. Likewise, cooking methods were found to significantly affect the total polyphenol content on kale, white cabbage, red radish, beet, black radish, turnip, red cabbage and broccoli. All vegetables except kale and white cabbage lost their polyphenols after boiling and red radish recorded the highest loss followed by beet, black radish, turnip, red cabbage and broccoli. On the other hand, blanching in boiling water increased the TPC of kale and white cabbage. Lastly in an investigation of the antioxidant properties of tomatoes after processing, it was revealed that boiling and baking had a small effect on the TPC of the tomatoes while frying gave a significant decrease of the TPC [23].

### Effect of cooking time on total phenolic content and total flavonoid content

For increasing cooking times, TPC differed for both aqueous and ethanolic extracts. As per ANOVA test, these differences were not significant for both aqueous extracts (*p* = *0.511*) and ethanolic extracts (*p* = *0.380*). However, TFC differed for aqueous extracts, and for ethanol extracts, it decreased with increase in the time of cooking. Overall, time did not have a significant effect on the TPC and TFC of the garlic extracts. This result is concordant with the observation of [25] who reported that cooking time did not significantly affect the TPC and antioxidant activity of edible mushrooms. However, the cooking time considered (≤ 5 min) was too short to significantly affect the TPC and antioxidant activity. Conversely, Hwang et al. [12] investigating the effect of different cooking methods and time (5, 10, 15 min) on the content and antioxidant activity of red pepper found that boiling reduced the TPC (13.9 to 54.9%) while prolonged cooking decreased the content of red pepper.

### Effect of cooking temperature and time on phytochemicals and total in vitro antioxidant activity of garlic

As the cooking temperatures were increased, the antioxidant activity also increased for the aqueous extracts. However, the antioxidant activity of the ethanolic extracts decreased with increase in temperature. Table 2 shows that the antioxidant activity of aqueous and ethanolic extracts were significantly affected by boiling at higher different temperatures. Thus, the antioxidant activity of garlic extracts increased significantly with the increase in temperature. On the other hand, the antioxidant activity also increased with increase in cooking time, but the differences were insignificant for both extracts. Previous authors recorded different observations while processing vegetables. Jim´Enez-Monreal et al. [26] evaluated the influence of cooking methods (boiling, microwaving, pressure-cooking, griddling, frying, and baking) on the antioxidant potential of vegetables and concluded that boiling was one of the methods that caused significant reduction in the antioxidant activity. It was reiterated that garlic (one of the vegetables studied) maintained its antioxidant activity even after boiling. This could be because according to the method used, garlic was cooked/boiled whole without crushing or chopping. It is therefore possible that the bioactive compounds responsible for the antioxidant activity were not activated or released hence the effect could not be properly accounted for. Further to that, Sutana et al. [27] argued that such differences could be as a result of the vegetables themselves (bioactive structures), the cooking method and the bioavailability of the phenolic compounds [26]. Similarly, Abacan et al. [25] in their study concluded that the DPPH scavenging activity of mushrooms decreased significantly (*p < 0.05*) when boiling temperature was increased. This result corresponded with the decrease in the TPC of the mushrooms as the temperature was being increased. The difference in the results could be due to the fact that, the phenolic compounds that leached in the cooking water were not taken into consideration as discussed earlier hence the decrease in TPC and antioxidant activity observed.

Generally, increase in temperature and cooking time increased the antioxidant activity of garlic. This is because temperature enhances the extraction of bound phenolic compounds thereby increasing the phytochemicals and antioxidant activity of garlic. This is true if the leached phytochemicals are considered, and provided decanting is not involved as a method of cooking.

## Limitation

Fractionation and characterization of the phenolic and flavonoid compounds in the extracts were not done. Further, isolation and characterization of phytochemicals at the different cooking temperatures and time were not done. Therefore, it is recommended that further studies should characterize the pure compounds from the garlic extracts so that the effects of temperature and time on these phytochemicals and their antioxidant activity will be well established.

## Supplementary Information

**Additional file 1: Table S1.** Raw data for the total phenolic content, total flavonoid content and antioxidant activity of aqueous and ethanol extracts of garlic boiled at different temperatures.

## Data Availability

The datasets supporting the conclusions of this study are included within the article (and its additional files).

## Abbreviations

FT-IR: Fourier Transform Infrared
TFC: Total flavonoid content
TPC: Total phenolic content
